# A Point Prevalence Study of Need and Provision of Palliative Care in Adult and Medical Surgical Inpatients

**DOI:** 10.1111/jocn.17775

**Published:** 2025-04-10

**Authors:** Alannah L. Cooper, Dipna Martin‐Robins, Natalie Panizza, Sally Coppock, Janie A. Brown

**Affiliations:** ^1^ School of Nursing College of Health & Education, Murdoch University Perth Western Australia Australia; ^2^ Royal Perth Hospital Perth Western Australia Australia; ^3^ School of Nursing Curtin University Perth Western Australia Australia; ^4^ St John of God Midland Public and Private Hospital Perth Western Australia Australia; ^5^ The Western Australian Group for Evidence Informed Healthcare Practice Curtin University Perth Western Australia Australia

**Keywords:** hospital‐based, inpatient, life‐limiting illness, need and demand, palliative care, prevalence, supportive care

## Abstract

**Aim:**

To gain an understanding of palliative care need and provision in adult medical and surgical inpatients.

**Design:**

An observational point prevalence study was conducted across four study sites in Western Australia.

**Methods:**

All data were collected directly from patient medical records by Registered Nurses. Potential palliative care need was assessed using disease‐specific indicators for the 12 conditions outlined in the Gold Standards Framework Proactive Indicator Guidance.

**Results:**

A total of 865 medical and surgical inpatients met study inclusion criteria. Across the four study sites, 38% (*n* = 331) of adult inpatients reviewed could have potentially benefitted from palliative care. Of the *n* = 331 patients assessed as having indicators for palliative care, there was evidence that 27% (*n* = 90) were currently receiving some form of palliative care, while 3% (*n* = 9) had been referred for specialist palliative care. For the majority of patients (70%, *n* = 232) there was no evidence of them receiving any form of palliative care or awaiting specialist palliative care.

**Conclusion:**

This study identified high levels of potential palliative care need among adult medical and surgical inpatients. The majority of the patients identified as having indicators for palliative care were not receiving any form of palliative care.

**Implications for the Profession and/or Patient Care:**

The high prevalence of palliative care need found in this study highlights that recognising and addressing palliative care is essential for high‐quality care for medical and surgical inpatients. To address the high level of need identified all nurses require basic palliative care training to provide optimal patient care.

**Impact:**

Knowledge about the level of palliative care need and provision of palliative care in public hospitals was limited. This study identified a high prevalence of potential palliative care need in medical and surgical inpatients. The majority of patients with indicators for palliative care were not receiving any form of palliative care. This research demonstrates that palliative care needs should be considered by all registered nurses and other health professionals caring for medical and surgical inpatients.

**Reporting Method:**

The study is reported using the STROBE guidelines.

**Patient or Public Contribution:**

No patient or public contribution.

**Summary**

- There is a high prevalence of palliative care need in medical and surgical inpatients that is largely unmet.
- All health professionals need to be able to recognise palliative care need and provide generalist palliative care.

## 1 Introduction

Palliative care is an active, holistic approach to the care of individuals with life‐limiting conditions that includes the management of physical symptoms, psychological distress, social and spiritual needs (Radbruch et al. 2020). Timely integration of palliative care can improve quality of life, reduce symptom burden, prolong survival and reduce healthcare costs (Gaertner et al. 2017; Janberidze et al. 2020). For patients to gain the maximum benefits of palliative care, the evidence suggests that at least three to four months of palliative care integration within the multidisciplinary team is needed (Davis et al. 2015). Despite the known benefits of early palliative care, delayed integration and non‐access remain a significant issue (Pitzer et al. 2024).

## 2 Background

A systematic review and meta‐analysis of the duration of specialist palliative care before death reported in research studies found a median duration of 18.9 days across the 23 countries included (Jordan et al. 2020). Patients in Australia received the fewest days of palliative care with a median duration of only six days. Even patients in Canada with the highest median duration of 69 days fell well below the three to four months minimum recommended integration of palliative care to optimise patient outcomes. National data sets drawn upon by the Australian Institute for Health and Welfare (AIHW) (2024) suggest that the median duration of specialist palliative care is 15 days before death. While the data from the AIHW (2024) has a longer median duration than the included Australian research studies in the review by Jordan et al., the 15‐day duration still falls well short of the recommended minimum.

As well as disparities in palliative care access between countries, there are also significant differences between settings (Bennett et al. 2016; Jordan et al. 2020) and disease types (Bennett et al. 2016; Jordan et al. 2020; Quinn et al. 2021). Patients who are referred as outpatients receive more days of palliative care, as do patients with cancer versus non‐cancer diagnoses (Bennett et al. 2016; Jordan et al. 2020; Quinn et al. 2021). National data from the AIHW (2024) mirror research findings, with Australian outpatients and patients with a cancer diagnosis receiving more days of specialist palliative care. Australian patients with cancer received an average of 31 days, patients experiencing organ failure received eight days and patients with frailty received six days.

The widespread delays reported in palliative care integration in the patient journey seem to be fuelled by misconceptions about palliative care. The term palliative care is often considered to relate specifically to end‐of‐life and terminal care (Guo et al. 2012; Kirkpatrick et al. 2017). Many health professionals and patients hold an incorrect belief that palliative care is only for patients in the end stages of life (Kirkpatrick et al. 2017). This leads to a failure to identify palliative care need and contributes to the delayed and late access to palliative care found internationally (Jordan et al. 2020).

Palliative care can be facilitated through a generalist palliative care approach from the patient's treating team, or through specialised palliative care services. Palliative Care Australia (PCA) (2018) advocates the provision of palliative care by all health professionals: ‘PCA's position is that palliative care is everyone's business. All health professionals who provide care to people living with a life‐limiting illness, their families and carers should have minimum core competencies in the provision of palliative care’. However, in practice many health professionals have limited knowledge and training in palliative care. Generalist palliative care should be provided by all health professionals to people who have life‐limiting conditions to address the bio‐psycho‐existential, social, cultural and spiritual needs of the patient and their family (PCA 2018; van Zuilekom et al. 2024). Additional support from specialist palliative care is needed when a patient has complex and persistent symptoms/needs and is provided by a multidisciplinary team with specialised skills, competencies, experience and training in palliative care (PCA 2018; Radbruch et al. 2020).

There is local and international evidence that large numbers of patients have unmet palliative care need (Cooper et al. 2021; Rosenwax et al. 2016; Szekendi et al. 2016). A point prevalence study of adult inpatient palliative care need conducted at a Western Australian private, not‐for‐profit hospital found that at a hospital population level, 29% (*n* = 78) of inpatients could have benefited from palliative care (Cooper et al. 2021). Of the 78 inpatients assessed as meeting criteria for palliative care, 29% (*n* = 23) were currently receiving palliative care, with a majority of patients (71%, *n* = 55) not receiving palliative care. The results demonstrated a large level of unmet palliative care need in this private inpatient population. While the results of this study are useful, they may not be generalisable to public hospital settings. To better understand the extent of palliative care need across hospital inpatient settings, the earlier point prevalence study was replicated in a cohort of medical and surgical inpatients admitted in Australian public hospitals in the same jurisdiction.

## 3 The Study

### 3.1 Aim and Objectives

The aim of this study was to gain an understanding of palliative care need and provision in adult medical and surgical inpatients across a Western Australian public health service. The objectives of the study were to:

1. Determine the size and characteristics of the population of adult inpatients with potential palliative care need across the study sites,
2. Establish what percentage of patients who were appropriate for palliative care were receiving palliative care or had been referred to specialist palliative care services at the study sites,
3. Compare referrals to specialist palliative care and access to palliative care across study sites and palliative care service models.

## 4 Methods

### 4.1 Design

Point prevalence studies were undertaken across four sites. The project initially collected data at study sites 1 and 2 on 27 June 2023 and then gained further funding to collect data at study site 3 on 25 July 2024 and study site 4 on 16 August 2024. All data were collected during winter to control for any potential seasonal variation when comparing data between sites.

### 4.2 Study Sites

*Study sites 1 and 2* are public hospitals with a total of 649 inpatient beds across the two sites. These sites are sister hospitals and have some shared services. There is a consultative model of palliative care servicing at study site 1. At the time of data collection there were staffing levels of approximately 1.6 full time equivalent (FTE) provided by three Palliative Medicine Specialists Consultants, 1 FTE provided by one Registrar, 1.8 FTE provided by two Nurse Practitioners, 1 FTE provided by one Clinical Nurse, 1 FTE provided by one Social Worker, 0.2 FTE provided by one Pharmacist and a non‐clinical 1 FTE provided by one Secretary. There is no formalised establishment to provide a palliative care service at study site 2. At the time of data collection, one of the two Nurse Practitioners from site 1 provided 0.2 FTE onsite at study site 2 once a week and also provided phone support to staff as needed. Patients can be referred by their treating Medical or Surgical teams to palliative care services. During 2023 there were 1583 referrals to the palliative care service. There are five outpatient clinics run by a consultant, the Registrar and Nurse Practitioners.

*Study site 3* is a public‐private partnership with a total of 196 medical and surgical inpatient beds and 56 mental health inpatient beds. There is an inpatient consultancy model of palliative care at the hospital with staffing levels of 0.5 FTE Palliative Medicine Specialist Consultant and 0.8 FTE Clinical Nurse Consultant at the time of data collection. During 2023 there were 612 referrals to the palliative care service.

*Study site 4* is a public hospital with a total of 170 medical and surgical inpatient beds and 41 mental health inpatient beds. There is a consultative model of palliative care for inpatients, with staffing levels of approximately 0.2 full‐time equivalent (FTE) provided by one Palliative Medicine Specialist Consultant and 1 FTE provided by two Nurse Practitioners at the time of data collection. During 2023 there were 495 referrals to the palliative care service.

### 4.3 Inclusion and Exclusion Criteria

Adult medical and surgical inpatients admitted on the nominated date for each study site were eligible for inclusion. Patients who were < 18 years old, patients in the emergency department, patients admitted to a mental health ward and patients admitted for a same‐day procedure such as day surgery or dialysis were excluded from the study.

### 4.4 Data Collection

**FIGURE 1:**
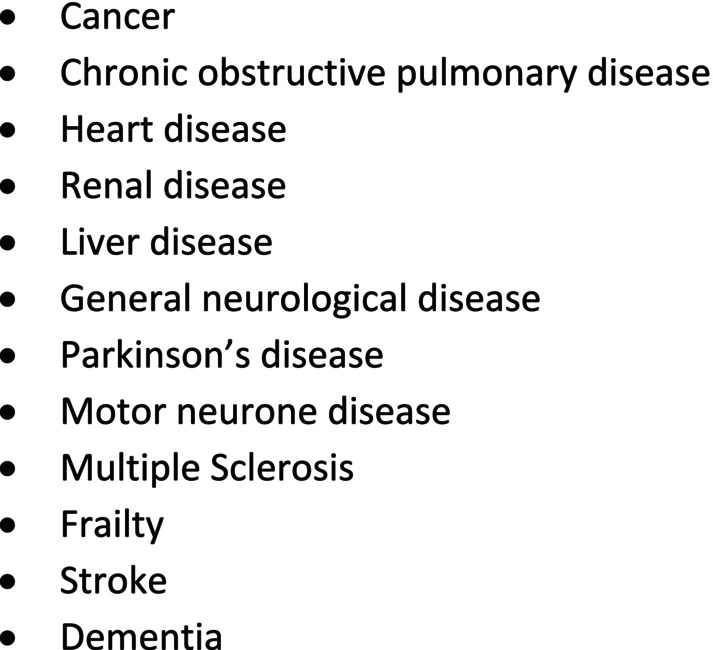
The 12 life‐limiting conditions included in the GSF PIG.

The data collection procedure was replicated from an earlier inpatient point prevalence study (Cooper et al. 2021). All data were collected directly from patient medical records by Registered Nurses who received study‐specific training prior to data collection to ensure consistency. A data dictionary was developed to provide clear definitions and support consistent data collection. The data dictionary included The Gold Standards Framework Proactive Indicator Guidance (GSF PIG) (Thomas and Armstrong Wilson 2016), an internationally recognised tool that assists clinicians to identify indicators for potential palliative care need. Disease‐specific proactive indicators of palliative care need are outlined for 12 life‐limiting conditions (Figure 1) included in the GSF PIG. Early identification of palliative care need is promoted by the GSF PIG with the use of the surprise question ‘Would you be surprised if the patient were to die in the next year, months, weeks, days?’

#### 4.4.1 Patient Characteristics

Data were collected on the characteristics of all patients that met study inclusion criteria, including age, biological sex, cultural identity, study site, primary treating speciality and history of any of the conditions outlined in the GSF PIG (Thomas and Armstrong Wilson 2016). For patients with no history of the conditions outlined in the GSF PIG data collection ceased.

#### 4.4.2 Assessment of Potential Palliative Care Need

For patients with one or more of the conditions outlined in the GSF PIG further information was extracted from their medical records for the items relevant to the conditions they experienced. This information was used to determine if the patient had sufficient proactive indicators for the condition(s) to suggest the patient may benefit from palliative care. For patients with potential palliative care need, data regarding any evidence of the patient receiving any form of palliative care were collected. In keeping with the original point prevalence study (Cooper et al. 2021), evidence of palliative care need being met was defined as a palliative care approach by the patient's primary treating team (generalist approach) that could include documented appropriate Goals of Patient Care, evidence of patient and/or family discussion around wishes for future care, advance care planning to clearly define treatment ceilings or referral to specialist palliative care services.

### 4.5 Data Analysis

Data were exported to Stata (StataCorp 2021) for analysis. Descriptive statistics were used to describe patient characteristics, level of palliative care need and the provision of palliative care across the four study sites.

### 4.6 Ethical Considerations

An application to undertake this research was submitted to the Royal Perth Hospital Human Research Ethics Committee and St John of God Health Care Human Research Ethics Committee. Approval was sought and gained (5782 & 2122) to conduct a point prevalence study with a waiver of consent to audit inpatient medical records (National Health and Medical Research Council, 2007). Reciprocal approval for the study was obtained from Curtin University (HRE2023‐0339).

## 5 Results

There were *n* = 866 medical and surgical inpatients across the four study sites. One patient was excluded from data collection as they were under 18 years of age. Of the *n* = 865 inpatients, data were collected on *n* = 428 admitted at study site 1, *n* = 94 admitted at study site 2, *n* = 197 admitted at study site 3 and *n* = 146 admitted at study site 4.

## 6 Collective Overview of Medical and Surgical Inpatients

### 6.1 Hospital Population Level Patient Characteristics

**FIGURE 2:**
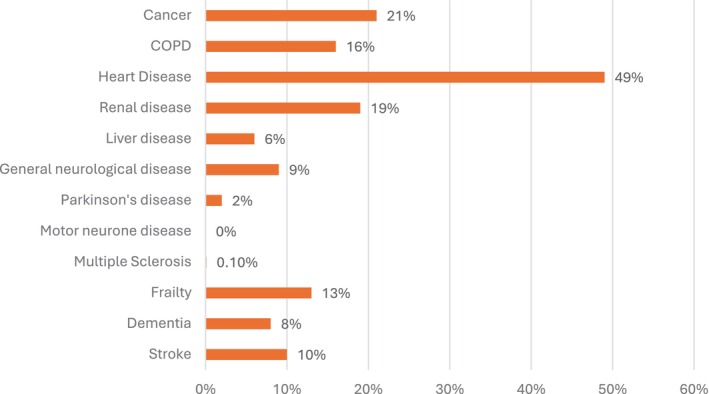
Proportion of patients with each of the conditions listed in the Gold Standards Framework. [Colour figure can be viewed at wileyonlinelibrary.com]

The sample was predominately male (54%, *n* = 470), with an age range of 18–100 and a mean age of 68 years. Data were available on the cultural identity of 766 patients; of these patients the majority identified as non‐Indigenous (91%, *n* = 696) while 9% (*n* = 68) identified as Aboriginal and < 1% (*n* = 2) identified as Torres Strait Islander people. Most patients (75%, *n* = 649) had at least one condition outlined in the GSF PIG. Heart disease was the most common life‐limiting condition (Figure 2).

### 6.2 Assessment for Potential Palliative Care Need

**FIGURE 3:**
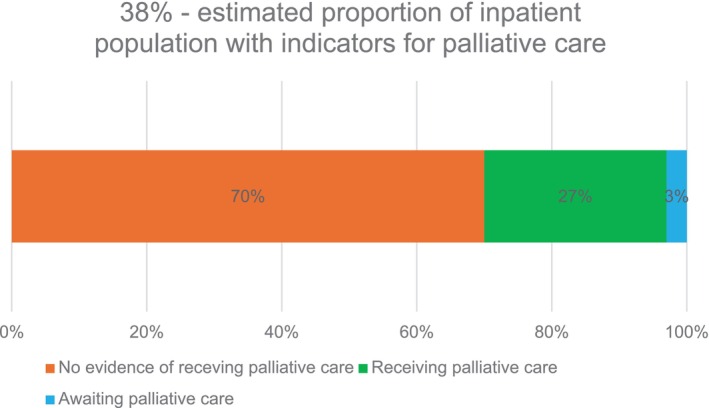
Prevalence of palliative care need and provision of palliative care. [Colour figure can be viewed at wileyonlinelibrary.com]

**FIGURE 4:**
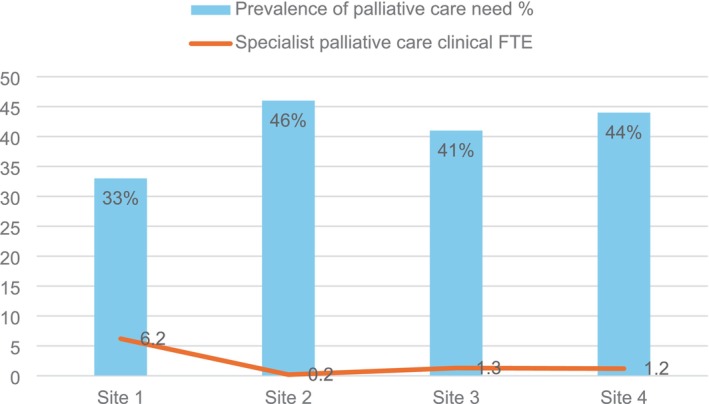
Prevalence of palliative care need and specialist palliative care staffing levels. [Colour figure can be viewed at wileyonlinelibrary.com]

Of the 649 patients with a life‐limiting condition listed in the GSF PIG, the response to the surprise question (would you be surprised if the patient were to die in the next year, months, weeks, days?) was ‘no’ for 57% (*n* = 371) of patients. Based on the review, 51% (*n* = 331) of patients with a condition listed in the GSF PIG could have potentially benefitted from palliative care. At a hospital population level, 38% (*n* = 331) of adult inpatients could have potentially benefitted from palliative care. Of the *n* = 331 patients assessed as having indicators for palliative care, there was evidence that 27% (*n* = 90) were currently receiving some form of palliative care, while 3% (*n* = 9) had been referred for specialist palliative care. For the majority of patients (70%, *n* = 232) there was no evidence of them receiving any form of palliative care or awaiting specialist palliative care (Figure 3). Of the *n* = 90 patients currently receiving palliative care, 57% (*n* = 51) were with involvement of specialist palliative care services and 43% (*n* = 39) of patients had evidence of a palliative care approach from their treating team. A comparison of the level of potential palliative care need and available specialist palliative care clinical FTE for each study site is presented in Figure 4.

## 7 Comparison of Patient Characteristics and Level of Need Across Study Sites

Based on mean age, study site 1 had the youngest patient cohort and the lowest level of potential palliative need of all of the study sites, with one‐third of patients at study site 1 identified as having indicators for palliative care (Table 1). The other three sites with older patient cohorts had a great percentage of patients with indicators for palliative care. Study site 2 had the highest mean age and the highest percentage of patients with indicators (Table 1). Nonetheless, high levels of unmet palliative care need were identified across all four study sites. Study site 2 had the lowest number of patients with indicators for palliative care having documented evidence of receiving any form of palliative care (Table 1).

**TABLE 1:** Comparison of patient characteristics and level of need across study sites.

	Study site 1 (*n* = 428)	Study site 2 (*n* = 94)	Study site 3 (*n* = 197)	Study site 4 (*n* = 146)
Female	40% (*n* = 170)	39% (*n* = 37)	54% (*n* = 106)	56% (*n* = 82)
Male	60% (*n* = 258)	61% (*n* = 57)	46% (*n* = 91)	44% (*n* = 64)
Mean age (range)	64 (18–100)	75 (18–99)	70 (22–98)	70 (28–97)
Identity				
Aboriginal	14% (*n* = 58)	4% (*n* = 4)	2% (*n* = 3)	2% (*n* = 3)
Non‐Indigenous	84% (*n* = 357)	92% (*n* = 86)	65% (*n* = 129)	85% (*n* = 124)
Torres Strait Islander	0% (*n* = 0)	1% (*n* = 1)	< 1% (*n* = 1)	0% (*n* = 0)
Not recorded	3% (*n* = 13)	3% (*n* = 3)	32% (*n* = 64)	13% (*n* = 19)
Patients with one or more condition included in GSF PIG	75% (*n* = 322)	65% (*n* = 61)	81% (*n* = 159)	73% (*n* = 107)
Patients with indicators of palliative need (hospital population level)	33% (*n* = 143)	46% (*n* = 43)	41% (*n* = 81)	44% (*n* = 64)
Patients with indicators and were receiving palliative care	26% (*n* = 37)	19% (*n* = 8)	30% (*n* = 24)	33% (*n* = 21)

## 8 Potential Palliative Care Need by Condition

The level of palliative care need and provision of palliative care was examined by life‐limiting conditions: patients with a history of one life‐limiting condition listed in the GSF PIG, patients with a history of cancer and other conditions listed in the GSF PIG and patients with a history of multiple conditions that did not include cancer (Table 2). The majority of patients experienced multiple life‐limiting conditions.

**TABLE 2:** Level of need by condition.

Condition(s)	Total number of patients	Patients with indicators of palliative care need	Patients with indicators receiving palliative care
Cancer only	32	53% (*n* = 17)	24% (*n* = 4)
COPD only	28	29% (*n* = 8)	38% (*n* = 3)
Dementia only	10	60% (*n* = 6)	33% (*n* = 2)
Frailty only	13	77% (*n* = 10)	10% (*n* = 1)
General neurological disease only	26	19% (*n* = 5)	0% (*n* = 0)
Heart disease only	97	12% (*n* = 12)	8% (*n* = 1)
Liver disease only	12	33% (*n* = 4)	0% (*n* = 0)
Parkinson's disease only	4	75% (*n* = 3)	67% (*n* = 2)
Renal disease only	15	33% (*n* = 5)	60% (*n* = 3)
Stroke only	7	29% (*n* = 2)	0% (*n* = 0)
Cancer and at least one other GSF PIG condition	145	74% (*n* = 108)	33% (*n* = 36)
Multiple GSF PIG conditions but not cancer	262	57% (*n* = 150)	26% (*n* = 39)

## 9 Palliative Care Need Based on Speciality

The level of palliative care need and referral rate was examined based on treating speciality. Specialities were split into two broad categories, those with a predominately medical focus and those with a predominately surgical focus. Patients with a medical treating team were more likely to have indicators for palliative care (Table 3). High levels of potential unmet need were evident in both medical and surgical patients. The level of palliative care need and evidence of palliative care provision based on the patient's primary treating team are presented in Table 4. Provision of palliative care could be via a palliative care approach from the patient's usual treating team alone or with support from the specialist palliative care service.

**TABLE 3:** Comparison of palliative care need for medical and surgical patients.

Speciality	Total number of patients	Patients with indicators of palliative care need	Patients with indicators receiving palliative care
Medical	589	45% (*n* = 267)	20% (*n* = 53)
Surgical	276	23% (*n* = 64)	20% (*n* = 13)

**TABLE 4:** Level of palliative care need based on primary treating team.

Speciality	Total number of patients	Patients with indicators of palliative care need	Patients with indicators receiving palliative care
Bariatric	3	0% (*n* = 0)	—
Cardiology	22	14% (*n* = 3)	33% (*n* = 1)
Colorectal	3	0% (*n* = 0)	—
Endocrinology	2	0% (*n* = 0)	—
ENT	4	0% (*n* = 0)	—
Gastroenterology	14	36% (*n* = 5)	0% (*n* = 0)
General medicine^a^	282	48% (*n* = 136)	32% (*n* = 44)^a^
General surgery	94	26% (*n* = 24)	17% (*n* = 4)
Geriatrics^b^	174	47% (*n* = 82)	34% (*n* = 28)^b^
Gynaecology	1	0% (*n* = 0)	—
Infectious diseases	4	25% (*n* = 1)	—
Intensive Care	6	100% (*n* = 6)	17% (*n* = 1)
Haematology	10	60% (*n* = 6)	17% (*n* = 1)
Neurology	32	28% (*n* = 9)	11% (*n* = 1)
Neurosurgery	7	14% (*n* = 1)	0% (*n* = 0)
Ophthalmology	3	0% (*n* = 0)	—
Orthopaedics	79	19% (*n* = 15)	33% (*n* = 5)
Plastics	21	14% (*n* = 3)	0% (*n* = 0)
Rehab medicine	8	0% (*n* = 0)	—
Renal	14	77% (*n* = 10)	0% (*n* = 0)
Respiratory	21	43% (*n* = 9)	22% (*n* = 2)
Trauma^c^	30	37% (*n* = 11)	36% (*n* = 4)^c^
Urology	16	25% (*n* = 4)	0% (*n* = 0)
Vascular	15	40% (*n* = 6)	0% (*n* = 0)

^a^ Four patients who have been referred for specialist palliative care services, so 35% (*n* = 48) where need was identified.

^b^ Four patients who had been referred for specialist palliative care services, so 39% (*n* = 32) where need was identified.

^c^ One patient had been referred for specialist palliative care services, so 45% (*n* = 5) where need was identified.

## 10 Discussion

This study aimed to gain an understanding of palliative care need and provision in four metropolitan Western Australian adult medical and surgical public hospital inpatients. Collectively, 38% (*n* = 331) of the adult inpatient population had potential palliative care need based on the proactive indicators outlined in the GSF PIG (Thomas and Armstrong Wilson 2016). The level of need in this study was higher than in an earlier point prevalence study conducted at an Australian private hospital, where 29% of inpatients had indicators of potential palliative care need (Cooper et al. 2021). Similar to other studies, there was evidence of a high level of unmet palliative care need, with the majority of patients with palliative care indicators receiving no form of palliative care (Cooper et al. 2021, 2024; Szekendi et al. 2016).

The higher level of palliative care need found in this study, compared to the earlier private hospital point prevalence study (Cooper et al. 2021), is reflective of the disparities reported across healthcare settings (Bennett et al. 2016; Jordan et al. 2020). Patients who attend public hospitals tend to have a lower socioeconomic status, experience higher levels of comorbidities, partake in riskier lifestyles and have lower levels of health literacy compared to patients who attend private hospitals (Jessup et al. 2018; Rana et al. 2020; Tynkkynen and Vrangbæk 2018). In Australia, private hospital admissions are predominately for planned elective procedures whereas public hospitals have a much larger proportion of unplanned admissions (Jessup et al. 2018), explaining the higher levels of acuity in public patient cohorts.

Disparities were also evident between the sites included in this study. The hospital with the highest level of unmet palliative care need, study site 2, had the lowest level of specialist palliative care services, with an onsite palliative care Nurse Practitioner providing service from study site 1 only one day a week. It was also evident that palliative care need was higher in the hospitals with a higher mean patient age. At study site 1 where there was the youngest patient cohort one‐third of the inpatients reviewed had potential palliative care need, and in the oldest patient cohort at study site 2 almost half of the patients had indicators.

As well as a high level of current potential palliative care need there was a high prevalence of patients with conditions outlined in the GSF PIG (Thomas and Armstrong Wilson 2016). This high prevalence is indicative of future need, given the progressive nature of the life‐limiting conditions included in the GSF PIG. Patients who did not have indicators of palliative care for the conditions they experienced at the time of data collection are likely to develop need as their disease progresses. Compounding this further was the finding that the majority of patients experienced multiple conditions included in the GSF PIG. These comorbidities represent a high level of acuity, complexity and risk in the patient cohort. This increasing acuity and complexity of patients is widely reported in other settings. Advances in medical treatments mean people with life‐limiting illnesses are living longer and there is a growing demand for palliative care as a result (Finucane et al. 2021; May et al. 2020).

The high prevalence of patients with indicators for palliative care in this study demonstrates the assertion by PCA (2018) that palliative care needs to be everyone's business. Ensuring all staff have basic palliative care training is essential for clinical practice and providing high‐quality patient care so that palliative care can be integrated early to optimise patients' quality of life, improve symptom control and reduce healthcare costs (Gaertner et al. 2017; Janberidze et al. 2020). With over a third of inpatients having indicators for palliative care need in this study, clinicians are constantly caring for patients with palliative care need. The volume of patients identified with potential palliative care need in this and other studies (Cooper et al. 2021; Rosenwax et al. 2016; Szekendi et al. 2016) highlights the need for generalist palliative care approaches to meet the needs of patients with relatively straightforward palliative care needs (PCA 2018; van Zuilekom et al. 2024). These generalist approaches are needed alongside specialist palliative care to manage patients with complex needs (PCA 2018; Radbruch et al. 2020).

### 10.1 Limitations

The study examines four sites in Western Australia so the generalisability of the results is limited and should be interpreted with this in mind. Data were collected based on the information available from inpatient medical records and assessed against the proactive indicators outlined in the GSF PIG. Given the variable quality of information recorded in medical records, this could lead to an under‐or over‐estimation of the level of palliative care need. The assessment conducted for this study does not reveal what proportion of patients with potential palliative care need had complex needs that would require specialist input or the proportion of patients with non‐complex needs that could be managed through a generalist approach.

### 10.2 Recommendations for Further Research

Future research is needed to identify the barriers to inpatient palliative care provision. Interventions that address the barriers to the provision of timely palliative care and improve access to generalist and specialist palliative care need to be developed and tested through co‐design.

### 10.3 Implications for Practice and Policy

The findings of this study highlight the prevalence of palliative care need in medical and surgical inpatients. Healthcare organisations need to take active steps to assess the operational requirements to address palliative care need. This includes training health professionals on how to recognise palliative care need, provide generalist palliative care to patients with non‐complex needs and seek support from specialist palliative care for patients with complex needs. Ensuring there are sufficient specialist palliative care staff to support patients with complex palliative care need is also essential.

## 11 Conclusion

This study identified high levels of potential palliative care need among adult medical and surgical inpatients. The majority of the patients identified as having indicators for palliative care were not receiving any form of palliative care. These failures to recognise and address palliative care need are likely to result in reduced quality of life and poorer symptom control for patients. Generalist and specialist approaches are needed to improve access to palliative care and ensure patient care is optimised.

## Author Contributions

Each named author has substantially contributed to conducting the underlying research and developing or reviewing this manuscript. Additionally, to the best of our knowledge, the named authors have no conflict of interest, financial or otherwise.

## Conflicts of Interest

The authors declare no conflicts of interest.

## Data Availability

The data that support the findings of this study are available from the corresponding author upon reasonable request.
